# Health seeking behaviours among electronic waste workers in Ghana

**DOI:** 10.1186/s12889-015-2376-z

**Published:** 2015-10-16

**Authors:** Emmanuel Asampong, Kwaku Dwuma-Badu, Judith Stephens, Roland Srigboh, Richard Neitzel, Niladri Basu, Julius N. Fobil

**Affiliations:** Department of Social & Behavioural Sciences, School of Public Health, University of Ghana, Accra, Ghana; Medical Unit, Ghana Post Company Limited, Accra, Ghana; Department of Biological, Environmental & Occupational Health Sciences, School of Public Health, University of Ghana, Accra, Ghana; La General Hospital, Accra, Ghana; Department of Environmental Health Sciences, School of Public Health, University of Michigan, Ann Arbor, USA; Faculty of Agricultural and Environmental Sciences, McGill University, Montreal, Canada

**Keywords:** Agbogbloshie, Ghana, Health, Behaviour, E-waste, Health insurance

## Abstract

**Background:**

Electronic waste workers are prone to various illnesses and injuries from numerous hazards thus the need for them to seek health care. The aim of this study was to describe health-seeking behavior, and social and other factors affecting this behavior, among electronic waste workers at Agbogbloshie, Accra, Ghana.

**Methods:**

In-depth interviews were conducted and analyzed qualitatively from a grounded theory perspective.

**Results:**

Workers experienced various kinds of ailments. These included physical injuries, chest and respiratory tract associated symptoms, malaria, headaches, body pains and stomach discomfort. They reported seeking health care from multiple sources, and the main determinants of health seeking behaviour were severity of illness, perceived benefit of treatment, accessibility of service, quality of service, ease of communication with service provider and cost of health care.

**Conclusion:**

Multiple sources of health care were used by the e-waste workers. As cost was a major barrier to accessing formal health care, most of the workers did not subscribe to health insurance. Since enrollment in health insurance is low amongst the workers, education campaigns on the need to register with the National Health Insurance Scheme would facilitate access to formal health care and could result in improved health outcomes among e-waste workers.

## Background

In many developing countries, the demand for information and computer based technology has largely been satisfied through the importation and shipment of used electronic products [1]. Consequently, there has been a surge in electrical and electronic waste (e-waste) materials in the waste stream of these countries [2–4]. In Ghana, the widespread inability to afford new electronic products, coupled with the quest to keep pace with the global advancement in electronic technology, has led to massive importation of mostly second-hand electronic gadgets from developed countries, primarily from Europe and North America [5, 6]. Studies have shown that where e-waste exists, informal recycling activities are performed under dangerous conditions [7–10].

Ghana’s e-waste dump at Agbogbloshie is reported to be the biggest in sub-Saharan Africa, one of the largest worldwide, and has thus attracted the attention of many international environmental groups, researchers, and journalists [11]. The dump is currently a site for trade in products recovered from the waste stream [12, 13]. Informal workers scavenge the waste, dismantle the scrap in proximity to open-air burning to recover precious components such as gold, copper, silver, aluminum, iron, and brass for sale [14]. The livelihood of many people depends on the income generated from these activities [15, 16].

It is important to note that there are socioeconomic and cultural dynamics that come to play within such an informal work environment and these can be burdensome for individuals involved in such work activities, the family and the community health as a whole. In addition, there are issues of poor living arrangements and hazardous working conditions. In spite of the dangers associated with this type of work, the high job demands and the associated low wages result in an effort-reward imbalance that creates dissatisfaction among these e-waste workers.

In Ghana, e-waste workers are prone to various illnesses and injuries from numerous hazards [17]. Some of the conditions may be subclinical and asymptomatic and may only be detected incidentally during clinical investigations. Non-use of formal channels of health care [18] may result in delay in diagnosis of some of these medical conditions with detrimental effects. Also in the event of injuries, refusal to seek appropriate medical care could make such individuals prone to a number of ailments [19] such as severe wound infections, poor wound healing, deformities associated with poor management of wounds and the risk of tetanus infection.

Reasons for health seeking behavior are varied among individuals in times of ill health [20]. Barriers to formal quality health care can therefore be removed to partly motivate vulnerable workers [21] in the informal sector such as e-waste workers to have access to quality and affordable health care that will help in the early diagnosis and adequate treatment of diseases and injuries. This study therefore sought to describe health-seeking behavior, and social and other factors affecting this behavior, among e-waste workers.

### Theoretical background

This study draws on the health belief model (HBM), a useful tool in understanding and predicting health care seeking behaviour [22] to explain the findings. The model proposes that health-related behavior depends on an individual’s perception of four critical areas; severity of a potential illness, susceptibility to that illness, benefits of taking a preventive action and barriers to taking that action. The assumption therefore is that health seeking behaviour is influenced by certain cognitive variables as well as established mechanisms to minimize the occurrence of disease within the social system.

HBM suggests that individuals are faced with alternative actions but usually choose one that is most likely to yield positive outcomes [23]. Consequently, changes in health behaviour occur under these circumstances, and in this case, e-waste workers are likely to seek care in times of ill-health when they hold certain beliefs about that ill-condition. These beliefs include perceived susceptibility to a particular health problem, in this case – whether the e-waste workers are at risk of ill health, perceived seriousness or severity of the health condition and its social consequences, belief in effectiveness of the new behaviour as the right thing to do, cues to action such as witnessing the death or illness of colleague e-waste workers, perceived benefits of preventive action and barriers to taking action.

It is believed that the combined effect of these beliefs influences to a large extent the likelihood of an individual to perform the behavior. The HBM can therefore be said to explain the relationship between an e-waste workers’ beliefs and behaviour by providing a way to understand and predict how they behave as far as their health is concerned.

## Methods

### Design and participants

A qualitative survey was conducted among e-waste workers at the Agbogbloshie dumpsite in April 2014. A total of twenty e-waste workers were recruited to participate in the study. The key recruitment criterion was that individuals had at least one year of continuous work experience in the Agbogbloshie e-waste site [24–26]. Written informed consent was sought from all participants after translation of the participant information sheet for the study to them, explaining the rationale of the study, procedures involved, participant’s rights, issues of confidentiality, voluntary participation, risks and benefits and the right to withdraw at any time without any prejudice. Ethical clearance was obtained from the Ethical Review Committee of the Ghana Health Service, Research and Development Division and the IRB of Noguchi Memorial Institute for Medical Research (NMIMR) of the University of Ghana, Legon. Prior to the study, permission to conduct the study was sought from the leaders (recognized gatekeepers) of the e-waste workers at Agbogbloshie site.

Data were collected from volunteers who were recruited based on their availability, from randomly selected locations across the site to help ensure representation for the various work activities undertaken by the e-waste workers. The interviews were moderated at a conducive setting at the site, with the assistance of two expert interviewers who were native Dagomba and fluent in both Dagbani and English. These interviewers had vast experience in conducting interviews and had in the past been involved in data collection at the study site. The information was recorded with a digital recorder after seeking consent from each of the participants. While one moderated the interview sessions, the other took field notes. Each interview lasted about 45 min. Participants were interviewed broadly on subject characteristics (age, marital status, formal education, and family/community interactions), illness characteristics (nature of illness, severity of illness, perceived cause of illness, and expected benefits of treatment) and service characteristics (appeal, accessibility, quality, communication, and cost). The interviews ended after 20 participants had been interviewed when it was realized that saturation had been achieved [27, 28].

### Analysis

The two expert interviewers separately translated verbatim and transcribed all the recorded interviews into English in a word processing application. Additionally, they expanded the interview notes taken during the interview sessions. In a situation where there was disagreement between the two interviewers, they reviewed the transcripts and the original recordings until consensus was reached. The transcripts and expanded notes were stored as files and coded manually for textual analysis in accordance with the principles of grounded theory [27, 28] since we sought to generate a theory rather than to test a hypothesis. Coding was specifically done by placing blocks of text into various nodes based on the categories and subcategories. Using the categories, information was compared across the transcripts based on similar and contrasting views of health seeking behaviour among e-waste workers. The themes were illustrated with verbatim quotes and interpreted based on existing literature.

## Results

### Participants’ characteristics

All 20 participants were male e-waste workers who had worked at the e-waste site for a period between one and seven years. Their ages ranged between 18 and 32 years. All of them hailed from the Northern Region of Ghana, and spoke Dagbani as their primary language. Only three out of the 20 participants had senior high school education, the rest had received primary level education or no formal education at all.

### Ailments of the E-waste workers

E-waste recycling activities include collection, dismantling and burning of e-waste as well as selling recovered materials such as copper wires and transistors. Some of the workers at the site engaged in multiple activities on a daily basis. Each of these activities exposed them to health conditions such as physical injuries and self-reported chest pains or symptoms of respiratory tract infections. Some workers also complained of frequent malaria attacks, headaches, body pains and stomach discomfort.

*“When the smoke goes into you breathing becomes difficult. You feel pains in your chest and you cough. You can be doing the work in the fire and the fire can get you injured.”*

*“You start work in the morning and walk for long distances. You also sit from morning till evening so my waist. Sometimes I have pains all over the body particularly my arms, sometimes I go to bed and cannot sleep, sometimes too I sleep and waking up becomes difficult. I sometimes have stomach ache, head ache and I also feel dizzy. I can also get malaria, and chest pains.”*

Most of the workers believed that their ailments were a result of the constant exposure to the fire, smoke and garbage they encountered at the work site.

*“It is not good for humans to be on the refuse dump, but because of how our work goes we are in it all the time and this makes us sick. The smoke also makes us get all these illnesses. When they burn the tires, we are in it and the smoke gets into us”.*

*“The smoke is a very big problem for us and you will sit down all day, you will not get up, you just sit at one place and dismantle, you know the hammer has weight. We are really suffering in this work. It is not small”*

One worker was of the view that the frequent episodes of malaria that they experienced were due to their sleeping arrangements. Most of the workers slept in the open without any protective cloth. Consequently, they risked being bitten by mosquitoes. This was described by a worker who said:

*“We get sick because of the mosquito problem. Mosquitoes bite us every day. You know since we do not have a room to sleep in and only sleep outside that is what causes the malaria”*

Almost all the respondents expressed concerns about the number and types of injuries associated with their work. The activities that resulted in injuries were mainly dismantling electronic equipment and burning of the insulated wires. Dismantling was associated with a lot of injuries from the use of metal instruments such as hammers to break television and computer screens. This was reported to cause many of the injuries among the workers. A worker in his description of dismantling activity said:

*“You know when you are doing the dismantling, we use a hammer, you can get confused and the hammer can knock your hand. I can take a TV to break and take out the metal in it, and it cuts me, and when dismantling you might want to hit a metal with a hammer and you end up hitting your hand.”*

Similarly, burning was associated with injuries especially for those who do not take precautions to ensure that a fire is extinguished after the burning.

*“Some of the things that get us injured are that, you can go to burn copper and after that if you do not put out the fire or there is a hot material on the ground, you might not know and step on it.”*

### Health seeking practices

Health seeking practices among the interviewed e-waste workers varied widely. They resorted to using different sources of health care and sometimes the simultaneous use of multiple sources. The main heath seeking practices reported included self treatment, reliance on traditional medicine, use of local chemical/drug stores and the use of hospitals and clinics. The use of drug stores for instance, was common among the workers because these stores were geographically convenient, as described by a worker who said:

*“When we are sick, we do not go anywhere, we usually go to the drug store at Sikkens or Konkomba Yam Market to buy medicine. Unless you go and buy “Para” or some other medicine you won’t get well. On both occasions that I had an injury I went to the drug store to buy medicine.”*

The use of traditional medicine appeared to be popular among ill e-waste workers, especially when in their own estimation, the illness was not that serious. The choice of traditional medicine was mainly based on low cost.

*“If you are sick and it is not serious you can buy the traditional medicine, which cost 2cedis, 3cedis (*less than a dollar*) and take it. I like it because it is not costly and when you buy it they can tell you to put the medicine in porridge without sugar and drink or mix it with water and drink.”*

It was obvious that e-waste workers resorted to multiple sources of health care until the aim of realizing relief for their symptoms. That was the practice in situations when a particular health care service did not produce the desired treatment outcome and they were then compelled to turn to a different source.

*“If I am sick, I will go and buy “quinine tonic”. This will help me to vomit. If I am still not well then I will buy the traditional medicine to try before going to drug store and if it also does not work then I have to go to the hospital even though I have no money*.”

*“When we take in the traditional medicine and we are still not well, we then buy medicine from the drugstore.”*

There was also the phenomenon where multiple concurrent uses of different forms of health care were utilized among e-waste workers. The most common was the simultaneous use of traditional medicine and medicines from a drug store. This was expressed by a worker who said:

*“Sometimes we use the local treatment and the medicine from the drug store together so that the one which can work fast will cure the problem”*

### Determinants of seeking illness care

Determinants of health seeking varied depending on the type of health care chosen, but generally accessibility, severity of ailment, perceived benefit of treatment, quality of service, ease of communication with care provider and cost of care were the major determinants of the first choice of access of health care. The main reason for not seeking treatment for an injury or illness was the feeling that the condition was not serious enough to merit treatment. Consequently, in this situation, workers chose to stay at home till the condition improved.

*“Some of the sickness we go to the hospital that is the one that is too much or serious, for some too we buy the traditional medicine around here which is not costly and for some too we go to the drugstore to buy medicine.”*

*“If you are sick and it is not serious you can stay in the house and come to work the following day, that is if you become well then you come back.”*

#### Severity of injury

Self-reported treatment practices were associated with the belief that prevailing ailments were not serious and could be treated with medicines that were low in cost and could be accessed easily.

*“When I get injured and it is not serious I put “So-Klin” on the injury because of the dirt so as to make it clean. I also buy medicine and plaster to put on it from those who go around selling medicine.”*

*“When I am working and I get a cut, and it is not that serious, I sometimes put the oil in the metals on it to stop the bleeding.”*

In their view some conditions required treatment at the hospital. They emphasized that minor injuries did not require the use of a hospital but in times of severe injuries there was the need to go to hospital.

*“If whilst working we get a serious injury we would go to the hospital, and if it is a mild injury we would treat it ourselves.”*

*“It has been a while since I had a serious injury but the last one I had they took me to the hospital. I had a deep cut and at the hospital I was given medicine and injection”*

*“I will only go to the hospital with serious injuries, if the bleeding is not stopping, then I will take it there.”*

#### Cost of health care

For most of the participants, the motivation to use a particular source of care was driven by the cost. They admitted that they sometimes had no choice than to try the traditional medicine because of its low cost bearing in mind that they could not afford other forms of treatment.

*“Since we do not have money to go to the hospital, they will tell you to go and buy the traditional medicine. If you work and get like 5cedis you can save 2cedis and spend 3cedis, so in one month’s time you can get 20cedis or 15cedis. When you get hurt the only thing you can do is to buy the traditional medicine, because the 15 and 20cedis cannot take you to the hospital.”*

Comparatively, the cost of treatment from drug stores for instance was perceived to be lower than going to a hospital as expressed by a worker:

*“When am sick I do not go to the hospital, I go to the drugstore and buy medicine. That one is not expensive, if you go and tell them your head is aching they will get you paracetamol which is 50 pesewas, then you take it home and swallow and it is finished.”*

Though there was the view that hospital care was necessary in some situations, the main problem with accessing this service by the workers was the perceived cost of health care at these facilities. The cost of health care was therefore seen as the main barrier to accessing health care at the hospital even when it was necessary. This was expressed by a worker who said:

*“The hospital is expensive. You pay for everything…. I do not have money to go to hospital, if you go there and after treatment you are unable to pay for your bills what are you going to do?”*

#### Ease of communication

The ease with which they communicated with the health care provider influenced their choice of obtaining care. At the drug store for instance, they would describe their conditions and the attendant at the drug store after listening to them would dispense the appropriate medicine. In addition to selling medicines they also dressed wounds of the injured workers. In many cases, the e-waste workers had established good relationships with Chemical Shop owners (these are shops or stores that sell over the counter medication that do not require prescription). There was easy communication and they were able to tell them their problems without any difficulty.

*“I think the drug store is better, the one at Sikkens is where I use when I am sick. The attendant there will not ask you so many questions and you are free to tell him your problems. I think the drugstore is the one that can help, that is why I go there when I am sick.”*

Some of the workers had even established some form of personal relationships and credit arrangements such that they could take medicines and pay back later when they had recovered and made some income/savings from work. Such arrangements were not available at hospitals and clinics.

*“If the drugstore owner knows you, he may allow you to pay part and pay the rest later, but if he doesn’t know you, you will have to pay all.”*

*“If you do not have the money you cannot go to the hospital, if you tell them to give you the medicine and you will come back to pay the next day they will not agree.”*

#### Perceived benefits of treatment

The use of traditional medicine was typically for mild symptoms, especially when it was easily accessible, coupled with the ease of communication with provider. There was also the belief that if they were unable to receive a cure after going through all the treatment options then they had to go back home to their relatives and resort to native medicine emphasizing the perceived benefits of traditional treatment. This was illustrated by a worker who said:

*“If after hospital treatment we are still not well we go up north to our fathers. They will use the traditional medicine there to take care of us knowing that that was what our forefathers were using.”*

### Poor patronage of the national health insurance scheme

Given the prevalence of injuries and illnesses, patronization of the National Health Insurance Scheme (NHIS) would seem attractive to at least some of the e-waste workers. On the contrary, most of the workers were not active members of the NHIS even though they acknowledged the usefulness of bearing an NHIS card. Most of them had never registered for the membership cards, citing cost of registration (Twenty four cedis equivalent of about seven US dollars) as the main barrier to assessing the scheme.

*“I really need health insurance but I do not have the money, I need it very well but I cannot afford it”.*

*“I do not have the health insurance card. I do not have money to register; I have not been able to work to get that money”.*

Some who had registered with the scheme in the past did not have valid NHIS identification cards, having lost them through theft, or, fire or having left them behind in their villages in the North before migrating to Accra. They also cited cost as a reason for not pursuing re-registration or requests for new NHIS cards bringing to the fore the need for health education amongst the workers on issues related to NHIS. Below are excerpts of the conversation with workers on their NHIS cards:

*“Since I came here I have not had the money to do one. I used to have one when I was up north, but when I came here, it got stolen.”*

*“My health insurance card got burnt up. Now I do not have one.”*

*“I did my health insurance card but did not bring it down here, I left it up north.”* “*I lived with my father, and he travelled, he has his own room and I have mine, I kept my own (*health insurance card*) in his room and he locked his door when he travelled that is why I did not bring it here.”*

## Discussion

The findings of this qualitative study indicated that injuries and illnesses were major concerns among e-waste workers, with dismantling and burning being the main activities causing the majority of the injuries. Cuts and burns were common among participants, as has been reported in other studies on e-waste workers at Agbogbloshie [27, 29, 30]. Other commonly reported conditions were chest pains and respiratory symptoms attributed to work place factors. This is consistent with findings in China and Brazil where e-waste workers have made similar health complaints as a result of the e-waste activities they undertake [31, 32]. Self-reported chest and respiratory symptoms had also been indicated as common complaints amongst the e-waste workers [33, 34]. These symptoms could be as a result of exposure to smoke and fumes of metals such as lead and cadmium. The fumes from these substances could cause multiple disorders, organ malfunction or even cancers [33–37]. In this survey, most of the e-waste workers lived on site near their work area. E-waste sites are usually overcrowded coupled with poor living conditions that make them vulnerable to ill health conditions [37].

Most e-waste workers were involved in the activity only for a relatively short period of time, and often gave up the job due to the onset of health problems, consistent with other studies [38]. The effects of chronic exposure to chemicals arising from e-waste processing activities may not be immediately felt as long latencies can exist between exposure and adverse health outcomes. As such, and with increased activities at Agbogbloshie there is an increased need to improve worker access to health facilities for screening, early detection, and appropriate intervention. Our findings suggest that E-waste workers coming south to Accra from the Northern Ghana are typically very healthy upon arrival, but after a short period, illnesses likely resulting from their work activities make them go back to the North to seek treatment for their ailments. This is the trend in China that has been referred to as the “countryside exporting good health and re-importing ill health” [39].

With respect to health seeking practices, the workers accessed health care from multiple sources with a pattern of seeking health care from a succession of types of health provider until the problem was resolved. These included self treatment, traditional medicine, use of drug stores and use of formal health care services in hospitals or clinics. The use of Chemical Shops was the most predominant choice for health care.

Self treatment involved the use of self prescribed concoctions or substances obtained from herbal medicines during illnesses and injuries. Some of the self treatment practices included application of detergents and lubricants to wounds. Anecdotal information has it that these substances help wash off dirt from wound surfaces and also protect wounds from deterioration. Among the e-waste workers, they hold the opinion that these substances are effective for the purpose of their wounds being protected from other infections. However, they may contain chemicals that may be detrimental in the long term. Traditional medicine involved herbal preparations or natural remedies usually recommended by herbal practitioners or local medicine men. Chemical Shops were a popular choice for obtaining treatment as e-waste workers had the opportunity to talk to the attendants who would then dispense some medication to them. In addition, they were provided with services such as wound care and had built relationships where they could go for medication on credit and paid back later. Formal health care was health care obtained from private or government hospitals or clinics with a qualified clinician.

Though participants’ characteristics such as age, sex, marital status, educational level as well as income were important determinants of health seeking behaviour, the study focused on service and illness characteristics [38].

Determinants for health seeking varied across individuals depending on the type of health care desired, but generally service and illness characteristics were the most important determinants for first point of call at a health facility. Subsequent choice of health care was determined by perceived benefit of treatment, severity of the ailment, accessibility of the service, quality of service, ease of communication with provider and cost of health care. Severity of ailment was a very important characteristic of health seeking behaviour. The notion among the workers was that mild illnesses and injuries did not require hospital treatment and could be treated with other forms of treatment. However, this practice could result in delay in accessing adequate treatment and for that matter tended to hinder prompt treatment.

Some of the self treatment practices such as the use of detergents and lubricants on wounds were motivated by their perceived benefits. The low educational level of the workers must have influenced such perceptions, probably pointing to the need for health education to improve their health seeking practices.

Accessibility was an important factor in determining choice of health care. Self treatment, traditional treatment and treatment at the Chemical Shops were generally considered to be easily accessible whilst formal health care at hospitals and clinics were not. As found in one study in the Dangme West District of Ghana, herbal remedies were commonly used because of accessibility and cost effectiveness [40]. There were no formal health care facilities in the area and considering the amount of commercial activities taking place at the site and the population, health authorities may consider the provision of a facility onsite.

Quality of service was one of the service characteristics which predominantly determined health seeking behaviour especially the use of drug stores among the e-waste workers. Good quality service is a key motivation for repeat visit during subsequent health challenges.

Additionally, good communication and good inter-personal relationship between health service providers and clients were important factors that enabled the e-waste workers to freely express their concerns especially when they visited the Chemical Shops. The e-waste workers were able to discuss their health problems with the Chemical Shop owners, who in turn prescribed medicines. With the formal health care provider, however, it took time and potentially several visits for a patient to access a doctor. Besides, the discriminatory tendencies and mistrust of medical professionals deter most migrant workers from attending the hospitals [31].

The most important determinant of health seeking was cost of health care. E-waste workers resorted to self treatment or traditional medicine because of low cost of treatment. The Licensed Chemical shops/drug stores were also patronized instead of hospitals because of lower cost of their services. Cost has been cited as the main reason for self medication rather than lack of access to medical care in general [41]. Perceived high cost of health care at formal health care facilities were the main barriers to accessing formal healthcare by the e-waste workers. It has been reported that cost and perceived quality of health care were the most important factors in health decision making [42]. Despite the fact that cost was a major barrier in accessing formal health care by the workers, the level of NHIS patronage was very low as well. Studies have shown that the high cost of health services and the lack of health insurance have made e-waste workers not patronize health care services from regulated clinics where they can be assured of supervised treatment [43]. Most of the e-waste workers interviewed did not have a valid NHIS membership card. Cost was again cited as the main reason for non registration, non renewal or replacement of expired or lost membership cards. In effect, e-waste workers could not access the formal health care when they were ill because they would be expected to pay out-of-pocket health expenses [31] while evidence shows that individuals on NHIS are significantly more likely to visit clinics and seek formal health care when sick [44]. In order to obtain access to the formal health care, e-waste workers need education on the need for registration with the NHIS as well the importance of retaining their cards and being in good standing for good health promotion.

## Conclusion

The e-waste workers have various health care needs from injury, infections and stress. They use a multiple of avenues to solve their health problems depending on their perceived severity of the condition and their ability to pay. Self-treatment by seeking aid from Licensed Chemical Sellers and the use of herbal preparations are driven by their affordability, flexible terms of repayment, the language used, familiarity and the attitude of the providers among others. The physical access and quick service also influenced the choice of first point of care. They also practiced self referral albeit at the later stages of treatment. Their health seeking practices need to be enhanced on pertinent issues identified in this study. This includes education on stress reduction strategies targeted at the work related stress factors identified, as well as the use of personal protective gear which could reduce the rate and severity of injury. Education is also needed on their self treatment practices that could be harmful rather than beneficial especially with the materials used to dress wounds. Awareness must be created on the need for registration with NHIS as this could improve access to quality health care. Through all these activities, the perceived high cost of enrolling on the NHIS among e- waste workers could be corrected and the benefits of enrolment emphasized.

## References

[1] Qasim A (2011). Linking health with global production networks: the case of the personal computer industry.

[2] Dedrick J, Kraemer KL. Globalization of the personal computer industry: trends and implications. UC Irvine: Center for Research on Information Technology and Organizations; 2002. Accessed on January 5, 2015 at: http://www.escholarship.org/uc/item/6wq2f4hx.

[3] Robinson BH (2009). E-waste: an assessment of global production and environmental impacts. Sci Total Environ.

[4] Schmidt SW (2006). Unfair trade e-waste in Africa. Environ Health Perspect.

[5] Robinson BH (2009). E-waste: an assessment of global production and environmental impacts. Sci Total Environ.

[6] Schmidt SW (2006). Unfair trade e-waste in Africa. Environ Health Perspect.

[7] Medina M (2005). Serving the unserved: informal refuse collection in Mexican cities. Waste Manage Res.

[8] Medina M (2008). The informal recycling sector in developing countries. Gridlines.

[9] Mitchell CL (2008). Altered landscapes, altered livelihoods: the shifting experience of informal waste collecting during Hanoi’s urban transition. Geoforum.

[10] Noble E (2008). E-waste think tank: review and synthesis.

[11] Agbogloshie, West Africa’s biggest e-waste dump [Internet]. Crikey Company; 2012 [cited 2012 Feb 3]. Available from: http://www.crikey.com.au/2012/01/31/e-dump-in-agbogloshie-west-africa.

[12] Johnson NM, Afriyie-Gyawu E, Huebner H, Marroquin-Cardona A, Robinson A, Tang L, Xu L, Ankrah NA, Ofori-Adjei D, Jolly PE, Williams JH, Wang JS, Phillips TD (2009). PAH exposure in a Ghanaian population at high risk for aflatoxicosis. Sci Total Environ.

[13] Ghana e-waste country assessment: SBC e-waste Africa project [Internet]. GreenAd-Empa; 2011 [cited 2012 Jul 13]. Available from: http://ewasteguide.info/files/Amoyaw-Osei_2011_Green Ad-Empa.pdf.

[14] Gabel DA. E-waste dump in Africa contaminating community. Global Pollut Prev News. [Internet] 2011 [cited 2012 Aug 27]; 2011(2011):2. Available from: http://isp.unu.edu/news/2011/media/files/Environmental_News_Network_Nov2011.pdf.

[15] Scheinberg A, Anschu¨ tz J. Slim Pickin’s — scavengers and waste pickers in the modernisation of urban waste management systems in the south. Waste: the social context; 2005 [cited 2013 Jan 14]. p. 553–6. Available from: http://connection.ebscohost.com/c/articles/36621215/slim-pickinsscavengers-waste-pickers-modernisation-urban-waste-management-systems-south

[16] Scheinberg A, Anschu¨ t J (2007). Slim pickin’s: supporting waste pickers in the ecological modernisation of urban waste management systems. Int J Technol Manage Sustain Dev.

[17] Akormedi M, Asampong E, Fobil JN (2013). Working conditions and environmental exposures among e-waste workers in Ghana. Int J Occup Environ Health.

[18] Amankwa EF (2014). E-waste Livelihoods, Environment and Health Risks: Unpacking the Connections in Ghana. West Afr J Appl Ecol.

[19] Rushton L (2003). Health hazards and waste management. Br Med Bull.

[20] Thomas J, Borrayo E (2011). An examination of moderators of perceived stress and illness behaviour. Psychology.

[21] Smedley BD, Stith AY, Nelson AR (2002). Unequal Treatment: Confronting Racial and Ethnic Disparities in Health Care.

[22] Jegede AS (1998). African Culture and Health.

[23] Munro S, Lewin S, Swart T, Volmink J (2007). A review of health behaviour theories: how useful are these for developing interventions to promote long-term medication adherence for TB and HIV/AIDS?. BMC Public Health.

[24] Oberhauser AM, Hanson KT (2007). Negotiating livelihoods and scale in the context of neoliberal globalization: perspectives from Accra, Ghana. Afr Geograph Rev.

[25] Oberhauser AM, Yeboah MA (2011). Heavy burdens: gendered livelihood strategies of porters in Accra, Ghana. Singapore J Trop Geogr.

[26] Twumasi-Ankrah K (1995). Rural–urban migration and socio-economic development in Ghana: some discussions. J Soc Dev Afr.

[27] Akormedi M, Asampong E, Fobil JN (2013). Working conditions and environmental exposures among e-waste workers in Ghana. Int J Occup Environ Health.

[28] Qualitative Research methods: a data collector’s field guide. Parkdatabase; 2005 [cited 2012 May 23]. Available from: http://www.fhi360.org/sites/default/files/media/documents/Qualitative%20Research%20Methods%20-%20A%20Data%20Collector's%20Field%20Guide.pdf

[29] Binion E (2012). The perception of health with informal recyclers in Buenos Aires, Argentina [dissertation].

[30] Prakash S, Andreas M, Obed A, Opoku, Amoyaw-Osei, Yaw; Mathias S, Esther M, Raphael F. Informal E-Waste Recycling Sector in Ghana: 2010; An Indepth Socio-Economic Study.

[31] Peng Y, Chang W, Zhou H, Hu H, Liang W (2010). Fcators associated with health-seeking behaviour among migrant workers in Beijing, China. BMC Health Serv Res.

[32] Gutberlet J, Baeder AM, Pontuschka NN, Felipone SNM, Dos Santos TLF (2013). Int J Environ Res Public Health.

[33] Feldt T, Fobil JN, Wittsiepe J, Wilhelm M, Till H, Zoufaly A, Burchard G, Göen T (2014). High levels of PAH-metabolites in urine of e-waste recycling workers from Agbogbloshie, Ghana. Sci Total Environ.

[34] Haefliger P, Mathieu-Nolf M, Lociciro S, Ndiaye C, Coly M, Diouf A, Neira M (2009). Mass lead intoxication from informal used lead-acid battery recycling in Dakar, Senegal. Environ Health Perspect.

[35] Hellstrom L, Elinder CG, Dahlberg B, Lundberg M, Jarup L, Persson B, Axelson O (2001). Cadmium Exposure and End-Stage Renal Disease. Am J Kidney Dis.

[36] Public Health Service National Toxicology Program. Report on Carcinogens 2005; (11 ed.). U.S.: Department of Health and Human Services.

[37] Hesketh T, Ye XJ, Li L, Wang HM (2008). Health status and access to health care of migrant workers in China. Public Health Rep.

[38] Kroeger A (1983). Anthropological and Socio-medical health care research in developing countries. Soc Sci Med.

[39] Zhang ZQ, Zhou Y, Lu SX, Chen YH (2007). Return migration of rural laborer from western China: causes and strategies. Stat Res.

[40] Asase A, Akwetey GA, Achel DG (2010). Ethnopharmacological use of herbal remedies for the treatment of malaria in the Dangme West District of Ghana. J Ethnopharmacol.

[41] Donkor ES, Tetteh-Quarcoo PB, Nartey P, Agyeman IO (2012). Self-medication practices with antibiotics among tertiary level students in Accra, Ghana: a cross-sectional study. Int J Environ Res Public Health.

[42] Russel S. Demand-Side Factors Affecting Health Seeking Behaviour in Ghana. George Town Undergrad Afr J Health Sci. 2008; 5(1).

[43] Blanchet NJ, Fink G, Osei-Akoto I (2012). The Effect of Ghana’s National Health Insurance Scheme on Health Care Utilisation. Ghana Med J.

[44] Hong Y, Li XM, Stanton B, Lin DH, Fang XY, Rong M, Wang J (2006). Too costly to be ill: Health care access and health seeking behaviours among rural-to-urban migrants in China. World Health Popul.

